# Ready, Set…Poised!: Polycomb target genes are bound by poised RNA polymerase II throughout differentiation

**DOI:** 10.15252/msb.20177968

**Published:** 2017-10-23

**Authors:** Alvaro Rada‐Iglesias

**Affiliations:** ^1^ Center for Molecular Medicine Cologne (CMMC) University of Cologne Cologne Germany; ^2^ Cologne Excellence Cluster for Cellular Stress Responses in Aging‐Associated Diseases (CECAD) University of Cologne Cologne Germany

**Keywords:** Chromatin, Epigenetics, Genomics & Functional Genomics, Genome-Scale & Integrative Biology, Transcription

## Abstract

In embryonic stem cells (ESCs), silent genes with major developmental functions display a unique epigenetic state in which strong and broad binding by Polycomb repressive complexes (PRCs) is accompanied by the presence of poised RNA polymerase II (RNAPII) and activating histone marks (e.g. H3K4me3) (Azuara *et al*, 2006; Bernstein *et al*, 2006; Stock *et al*, 2007; Brookes *et al*, 2012). It has been suggested that the plasticity and broad differentiation potential of pluripotent cells might rely, at least partly, on this unique epigenetic state (Bernstein *et al*, 2006; Stock *et al*, 2007). In their recent study, Pombo and colleagues (Ferrai *et al*, 2017) show that a similar epigenetic state can be found at a subset of major developmental genes throughout the differentiation of ESCs into neurons, providing novel and exciting insights into the molecular basis of cellular plasticity in differentiated cells.

Genes with major regulatory functions during the acquisition of specific somatic cellular identities are silent or expressed at low levels in ESCs. Accordingly, these genes are frequently embedded within particularly broad PRC‐bound domains. Interestingly, the promoter regions of these major cell‐identity genes were previously shown to be also occupied by H3K4me3, an activating histone mark, and were thus termed as *“bivalent”* promoters (Azuara *et al*, 2006; Bernstein *et al*, 2006). Subsequently, these *“bivalent”* genes were shown to be occupied by a unique form of RNAPII, called *“poised”* RNAPII, in which RNAPII is phosphorylated on Ser5 (S5p) but not on Ser7 (S7p) or Ser2 (S2p) (Stock *et al*, 2007; Brookes *et al*, 2012). It has been suggested that the presence of bivalent chromatin and poised RNAPII at these developmental genes might prime their subsequent activation during ESC differentiation (Bernstein *et al*, 2006; Stock *et al*, 2007). Consequently, it has been also hypothesized that the broad differentiation potential of pluripotent cells might depend, at least partly, on the unique epigenetic features that major developmental genes display in these cells. However, previous work demonstrated that “bivalent” genes could also be found in differentiating as well as fully differentiated cells, putting into question the relevance of this chromatin state for pluripotency (Mohn *et al*, 2008). Nevertheless, whether the presence of poised RNAPII at these genes was exclusive to pluripotent cells or, alternatively, also found in other cellular states has not been explored. Importantly, using a robust differentiation system in which ESCs are differentiated into post‐mitotic dopaminergic neurons, Ferrai *et al* (2017) now show that a subset of silent genes with major functions in the establishment of non‐neuronal cellular identities are continuously and simultaneously occupied by PRCs and poised RNAPII throughout differentiation (Fig 1). However, it is worth mentioning that, similar to what was previously reported for *“bivalent”* genes (Bernstein *et al*, 2006), the fraction of PRC target genes that are also bound by poised RNAPII is significantly larger in ESCs than in differentiated cells. Therefore, it is still possible that the pervasiveness of poised RNAPII/PRC genes in ESCs contributes to their pluripotency.

**Figure 1:**
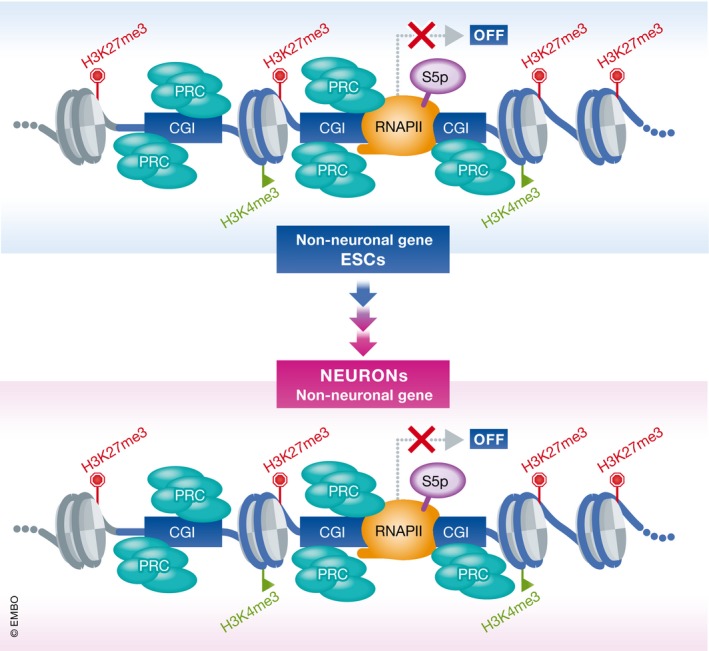
Polycomb target genes involved in differentiation towards non‐neuronal lineages are bound by poised RNAPII in differentiated neurons Silent genes with major developmental functions are bound in ESCs by PRCs and poised RNAPII. Moreover, these genes are marked by both activating (H3K4me3) and repressive (H3K27me3) histone modifications. Ferrai *et al* (2017) now demonstrate that such epigenetic state is found throughout differentiation of ESCs into neurons at a subset of Polycomb target genes with major regulatory functions in non‐neuronal cell lineages. PRC, Polycomb repressive complex; CGI, CpG islands; RNAPII, RNA polymerase II; S5p, serine 5 phosphorylation.

One major question immediately arising from the recent findings by Ferrai *et al* (2017) is whether the presence of poised RNAPII at a subset PRC target genes confers these genes with any distinct regulatory properties. Interestingly, Ferrai *et al* (2017) also show that the subset of PRC target genes occupied by poised RNAPII are especially sensitive to the loss of PRC silencing activity, becoming derepressed in both ESCs and differentiated neurons. It is worth mentioning though that the magnitude of the gene expression changes observed upon loss of PRC activity tends to be rather minor, in agreement with recent reports suggesting that PRCs might respond to, rather than impose, gene silencing (Riising *et al*, 2014). Nevertheless, Ferrai *et al* (2017) also uncover that poised RNAPII/PRC genes in differentiated neurons are particularly enriched in master regulators of non‐neuronal genes that are predicted to act as major drivers of trans‐differentiation towards non‐neuronal fates (e.g. heart, bone). Based on these observations, Ferrai *et al* (2017) postulate that the existence of poised RNAPII/PRC genes in differentiated cells might confer them with certain plasticity that can facilitate their trans‐differentiation into other cell types. Although it is questionable whether trans‐differentiation exists and/or plays any meaningful role under normal physiological conditions, it is clear that trans‐differentiation can be experimentally induced *in vitro* as well as occur under pathological conditions (e.g. cancer) (Vierbuchen *et al*, 2010). Therefore, if the poised RNAPII/PRC state indeed confers trans‐differentiation potential, then its manipulation might either promote or block trans‐differentiation, which can have important implications for both regenerative medicine and tumour biology.

Last but not least, another major open question that Ferrai *et al* (2017) tried to address is why only a subset of PRC target genes are also bound by poised RNAPII. Compared to other PRC target genes, Ferrai *et al* (2017) found that those bound by poised RNAPII display particularly broad PRC‐bound domains, are generally hypomethylated and are covered by multiple CpG islands, which can act as PRC recruitment elements in vertebrates (Deaton & Bird, 2011). Remarkably, these are all genetic and epigenetic features characterizing major cell‐identity genes. On the other hand, none of these properties seem to explain why poised RNAPII is preferentially recruited to these genes. Interestingly, it has been previously shown that while CDK7 is responsible of RNAPII S5P and S7P, the unique poised RNAPII state (i.e. S5P only) depends, at least in ESCs, on ERK1/2 activity (Tee *et al*, 2014). Although it remains to be shown how ERK1/2 are recruited to poised RNAPII/PRC genes and whether ERK1/2 are also responsible of S5P in differentiated cells, an intriguing possibility is that poised RNAPII/PRC genes might only be present in cells (differentiated or not) in which ERK1/2 activity is high. If this is true, then pharmacological modulation of ERK1/2 activity could perhaps be used to modulate cellular plasticity with either regenerative or therapeutical purposes.

## Conflict of interest

The author declares that he has no conflict of interest.
